# An Adaptable Interface Conditioning Circuit Based on Triboelectric Nanogenerators for Self-Powered Sensors

**DOI:** 10.3390/mi9030105

**Published:** 2018-03-01

**Authors:** Yongshan Hu, Qiuqin Yue, Shan Lu, Dongchen Yang, Shuxin Shi, Xiaokun Zhang, Hua Yu

**Affiliations:** 1 Key Laboratory for Optoelectronic Technology and Systems, Ministry of Education of China, Chongqing 400044, China; 20113211@cqu.edu.cn (Y.H.); 20103160@cqu.edu.cn (S.L.); 20113210@cqu.edu.cn (D.Y.); 20160802007t@cqu.edu.cn (S.S.); 20160813100@cqu.edu.cn (X.Z.); 2 Key Laboratory of Fundamental Science of Micro/Nano-Device and System Technology, Chongqing University, Chongqing 400044, China; 3 College of Optoelectric Engineering, Chongqing University, Chongqing 400044, China; 4 Department of Electro-Mechanic Engineering, Chongqing College of Electronic Engineering, Chongqing 401331, China; 200923008@cqcet.edu.cn

**Keywords:** adaptable interface conditioning circuit, impedance matching, TENG, self-powered sensors

## Abstract

In order to solve the limited life problem of typical battery power supply, a self-powered method that is based on the environmental energy harvesting has emerged as an amazing power supply approach. The Tribo-electric-Nano-generator (TENG) has been widely studied because of its high efficiency, low fabrication cost, and high output voltage. However, low output power conversion efficiency has restricted its practical application because of its own extremely high output impedance. In order to match the high output impedance of TENG and increase the output power, this paper presents an adaptable interface conditioning circuit, which is composed of an impedance matching circuit, a synchronous rectifier bridge, a control circuit, and an energy storage device. In the impedance matching circuit, the energy loss of coupling inductance could be reduced by using the bi-directional switch to increase the frequency, and impedance matching circuit can be used to increase the output efficiency of TENG. Experimental results show that, in about 3.6 s, the storing capacitor voltage was basically stable at 5.5 V by using the proposed adapted interface conditioning circuit in this paper. The charging efficiency has increased by 50%.

## 1. Introduction

The use of wireless sensors and the wearable devices has developed rapidly in recent years. In traditional way, they are usually powered by standard batteries. However, this approach has become a bottleneck restricting the development of wireless sensors and the wearable devices because batteries usually become depleted within a relatively short timeframe, and the replacement or recharge of batteries can increase the cost. The self-powered method based on the environmental energy harvesting provides a new solution for power supply of wireless sensor nodes and wearable devices [1,2,3,4]. Environmental energy harvester has been used as a battery to power-up the wireless sensor node or the wearable device. Usually, the environmental energy is weak and discontinuous, such as vibration energy, so the high efficiency, low power consumption is essential [5] The triboelectric nanogenerator (TENG), first invented by Zhong Lin Wang et al. in 2012, is able to produce electrical output based on triboelectrification and electrostatic induction in response to an external mechanical input. Its fundamental physics and output characteristics can be attributed to the Maxwell’s displacement current [6,7]. Up to now, the area power density of TENGs has reached 313 W/m^2^ and their volume energy density has reached 490 kW/m^3^ [8]. When compared with other energy generators, the TENG has high efficiency, low fabrication cost, and high output voltage. These advantages make TENG more suitable for self-powered wireless sensors and the wearable devices. Some studies of TENG have shown that charging efficiency decays quickly after several charging cycles, the maximum voltage of the energy storage device is much smaller than the open-circuit voltage of the TENG, regardless of the energy conversion efficiency of the TENG [9]. Also, the TENG has poor load capacity because of its high output impedance. In order to convert the output voltage of the TENG to a stable DC voltage and increase the energy-storage efficiency, an interface conditioning circuit is essential. Some studies have shown that the DC-DC circuit is effective in working as a conditioning circuit that can stabilize the output voltage successfully [10,11]. However, maximizing the power extraction from TENG is still unresolved, so impendence matching is a major consideration in circuit design and also it is the most efficient way to increase the output power. The fully-integrated self-powered wireless sensor node contains TENGs, which is an interface conditioning circuit, energy storage elements, and load circuits (wireless sensor node).

The objective of this paper is to present an adaptable interface conditioning circuit based on triboelectric nanogenerator. Firstly, we will discuss the output characteristics of TENG. Then, analysis of the interface conditioning circuit will be done in detail. Finally, the experimental results will be discussed.

## 2. Modeling of TENG

**Figure 1:**
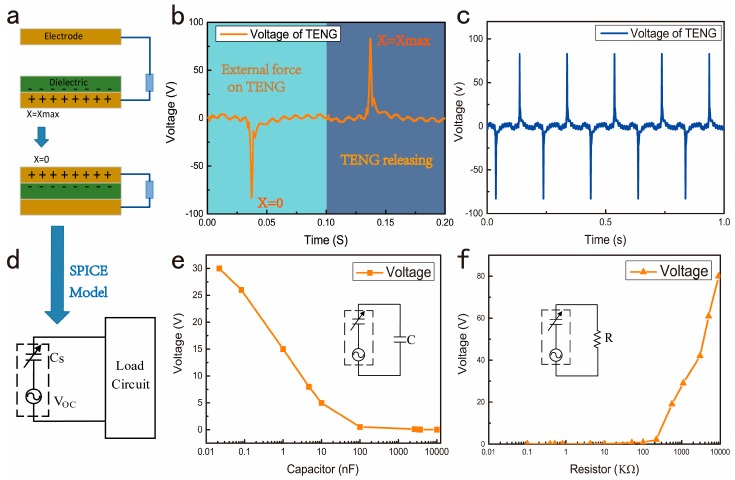
(**a**) Structure diagram of a Tribo-electric-Nano-generator (TENG). (**b**) Output voltage waveform of TENG in one working cycle without any load. (**c**) Open-circuit voltage of TENG. (**d**) Equivalent SPICE model of TENG. (**e**) Load voltage curve while the capacitor ranging from 22 pF to 10 μF is loaded. (**f**) Load voltage curve while the resistor ranging from 0.1 kΩ to 9.1 MΩ is loaded.

The fundamental working principle of TENGs is triboelectrification. Electrostatic induction is the main mechanism that converts mechanical energy to electricity. Any TENG contains two pairs of frictional electrified layers face to each other, the distance between the two layers is *X*. *X* will change when the external force applied, so the capacitance between the layers *C* will change. Triboelectric charges will be generated after the external force works, there will be induced charges between the two layers, as shown in Figure 1a. In order to get more charges, materials for triboelectrification is very important. According to the triboelectric series [8], polydimethylsiloxane and aluminum are selected as the materials for triboelectrification. In one contact- separation process, output voltage of TENG without any load is shown in Figure 1b, which reaches the maximum at *X* = 0 and *X* = *Xmax*. When we apply an external force on TENG periodically, output voltage is shown in Figure 1c. We can assume that the induced voltage between the two electrodes is *V*, transferred charges between the two layers is *Q*. The electrical potential difference *V* consists of two parts, one part is from the polarized triboelectric charges, and the other part is the already transferred charges *Q*, so *V* is given by the *V*-*Q*-*X* equation:

(1) $$V=-\frac{1}{C\left(X\right)}Q+V_{oc}\left(X\right)$$

Operating characteristics of TENG have been described in Equation (1), so we can get its inherent capacitive behavior from theoretical analysis [8,12,13]. From the *V-Q-X* equation of triboelectric nanogenerators, equivalent SPICE (Simulation program with integrated circuit emphasis) model can be easily obtained. As seen in Equation (1), there are two terms at the right side. Two circuit components are used to represent them. First, due to the inherent capacitance between the two electrodes of TENG, a capacitor $C_{s}$ is used to express its capacitance characteristics. The other term $V_{oC}\left(X\right)$ is an open circuit voltage term, which is resulting from the separation of the polarized tribo-charges and could be represented by a voltage source ($V_{oc}$). From these two terms, the whole equivalent SPICE model can be represented by a double-ended device that consists of an ideal voltage source a serial with a capacitor, as shown in Figure 1d. So, we can consider the TENG as a capacitive device in electrical analysis, and this is the reason why it has a poor load capacity. TENG’s output voltage is provided with different loads. In Figure 1e, the load voltage decreases as the load capacitance increases. In Figure 1f, the load voltage increases as the load resistance increases. The experimental results are consistent with the theoretical analysis.

Also, when we talk about a certain TENG, a theoretical calculation is an important method to get the characteristics of TENG, such as finite element method (FEM). Using FEM simulation software, such as COMSOL and ANSYS, the finite element calculation can be easily done. Another general method to get the characteristics of TENG is utilizing SPICE software, such as OrCad, Mulitisim. From the TENG equivalent SPICE model, the TENG can be obtained in theoretical calculation using SPICE software as a basic element consisting of a voltage source in serial connection with a capacitor [14]. After the motion process and the initial condition are generated, the powerful SPICE software can easily calculate the real-time output of any TENG systems.

When the signal source is used to drive a certain load, the voltage, current, total power consumption will change with the different load parameters. The main purpose of the impedance matching in the circuit is to adjust the load impedance, so that we can get the required optimal power with a certain power supply. To achieve the impedance matching in an AC circuit, the most effective way is to use the coupling inductances, the coupling inductances can not only achieve the impedance matching, but also regulate the output voltage [14].

**Figure 2:**
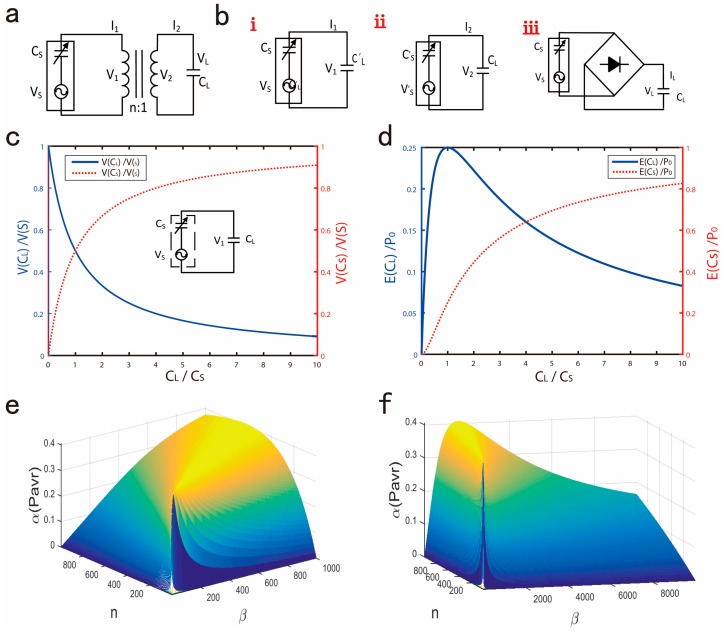
(**a**) Impedance matching circuit using coupling inductance. (**b**) **i**: Equivalent primary circuit of the coupling inductance; **ii**: The equivalent secondary circuit of the coupling inductance; **iii**: Charge a capacitor via a rectifier bridge. (**c**) Voltage curves of the $C_{L}$ and $C_{s}$ when $C_{L}/C_{s}$ changes from 1 to 10. (**d**) Power curves of the $C_{L}$ and $C_{s}$ when $C_{L}/C_{s}$ changes from 1 to 10. (**e**) Average charge efficiency diagram (*β* ranges from 0 to 1000). (**f**) Average charge efficiency diagram (*β* ranges from 1000 to 8000).

As shown in Figure 2a, we assume that the turns ratio of the coupling inductance is *n*, the load capacitance is $C_{L}$. So, we can get the equivalent circuit shown in Figure 2b. Figure 2bi shows the equivalent primary circuit of the coupling inductance. Figure 2bii shows the equivalent secondary circuit of the coupling inductance, the following equations are satisfied:

(2) $$C_{L}^{\prime }=n^{2}C_{L}$$

(3) $$C_{S}^{\prime }=\frac{C_{S}}{n^{2}},V_{S}^{\prime }=\frac{V_{S}}{n^{2}}$$

According to the above analysis, using the coupling inductance, the output voltage of TENG can be reduced to 1/n of the source output, the output current is increased by *n* times and output impedance is reduced to $1/n^{2}$. However, for an ideal capacitive load, it cannot extract energy from TENG because the average power of the load capacitor in one cycle is zero. When considering the load capacitance in one period is still very helpful. In Figure 2c, we assume that the load capacitance is $C_{L}$, the voltage extract from TENG is shown below:

(4) $$V\left(C_{L}\right)=\frac{C_{S}}{C_{S}+C_{L}}V_{S}$$

(5) $$E\left(C_{L}\right)=\frac{1}{2}C_{L}V_{L}^{2}=\frac{1}{2}C_{s}V_{s}^{2}\left(\frac{1}{2+\beta +1/\beta }\right)$$

Among them, $\beta =C_{L}/C_{s}$, define $P_{o}=\;\frac{1}{2}C_{s}V_{SMAX}^{2}$, so in half period, when $V_{s}$ gets the maximum value $V_{smax}$, the $V\left(C_{L}\right)$, $E\left(C_{L}\right)$, also get the maximum value, the curve of $V\left(C_{L}\right)$ and $E\left(C_{L}\right)$ are shown in Figure 2c,d. To prevent current reflow, we consider using a rectifier bridge, as shown in Figure 2biii. Assume that the voltage loss of the rectifier bridge is $V_{D}$. So, in the first period:

Initial value:

(6) $$V{\left(C_{L}\right)}_{0}=V{\left(C_{S}\right)}_{0}=0\;V_{S0}=V_{D}$$

During the charging process, the circuit KCL (Kirchhoff's current law) equation is:

(7) $$V_{S}=V\left(C_{S}\right)+V\left(C_{L}\right)+V_{D}$$

So, we can get the voltage $V\left(C_{L}\right)$, $\;V\left(C_{S}\right)$, shown below:

(8) $$V{\left(C_{L}\right)}_{1}=V{\left(C_{L}\right)}_{0}+\Delta V{\left(C_{L}\right)}_{1}=\frac{1}{1+\beta }\left(V_{SMAX}-V_{D}\right)$$

(9) $$V{\left(C_{S}\right)}_{1}=V{\left(C_{S}\right)}_{0}+\Delta V{\left(C_{S}\right)}_{1}=\frac{\beta }{1+\beta }\left(V_{SMAX}-V_{D}\right)$$

After *n* periods, we get $V{\left(C_{L}\right)}_{n}$, $P{\left(C_{L}\right)}_{n}$, shown below:

(10) $$V{\left(C_{L}\right)}_{n}=\;\left[1-\frac{\beta {\left(\beta -1\right)}^{n-1}}{{\left(1+\beta \right)}^{n}}\right]\left(V_{SMAX}-V_{D}\right)$$

(11) $$P{\left(C_{L}\right)}_{n}=\frac{1}{2}C_{L}V{\left(C_{L}\right)}_{n}^{2}=\beta [1-\frac{\beta {\left(\beta -1\right)}^{n-1}}{{\left(1+\beta \right)}^{n}}{]}^{2}P_{0}$$

Define that:

(12) $$P_{0}=\frac{1}{2}C_{S}{\left(V_{SMAX}-V_{D}\right)}^{2}$$

(13) $$P_{avr}\left(C_{L}\right)=\frac{P{\left(C_{L}\right)}_{n}}{n}\;=\;\frac{1}{n}\beta [1-\frac{\beta {\left(\beta -1\right)}^{n-1}}{{\left(1+\beta \right)}^{n}}{]}^{2}P_{0}$$

(14) $$\alpha \left(P_{avr}\right)=\frac{1}{n}\beta [1-\frac{\beta {\left(\beta -1\right)}^{n-1}}{{\left(1+\beta \right)}^{n}}{]}^{2}$$

*P*_0_ represents the maximum energy value that a single charge cycle can provide determined by the power supply, $P_{avr}\left(C_{L}\right)$ indicates average $P\left(C_{L}\right)$ in one cycle, so the parameter $\alpha \left(P_{avr}\right)$ is used to characterize the average charge efficiency. As shown in Figure 2e,f, for a certain *n*, $\alpha \left(P_{avr}\right)$ gets the maximum value corresponding to an optimal value of *β*, and for a certain *β*, $\alpha \left(P_{avr}\right)$ gets the maximum value corresponding to an optimal value of *n*. Consider the following two points: (a) capacitor leakage or capacitance capacity is limited; (b) charging time is limited. So, according to the limitations of the actual application, select the optimal *n* and *β*.

## 3. Results and Discussion

As described above, we have some understanding of the output characteristics of TENG and impedance matching, in this section we focus on the interface conditioning circuit. The following key design ideas for interface conditioning circuit must be taken into account, including: (a) maximizing power extraction from TENG by using the impedance match technology; (b) storing the harvested energy; (c) conditioning output voltage to meet the power requirements of the sensor node; and (d) minimizing power consumption of the whole system.

**Figure 3:**
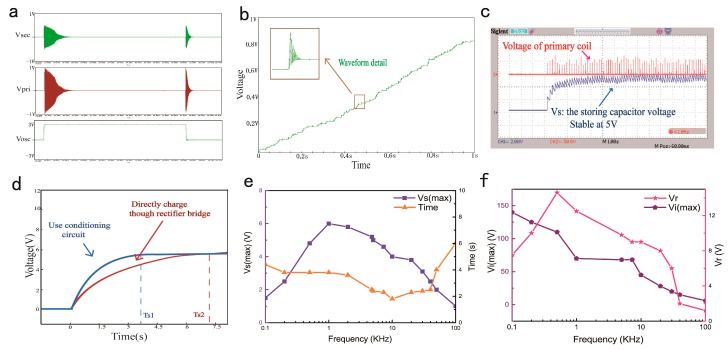
(**a**) Voltage simulation curves of the secondary inductance (*Vsec*), primary inductance (*Vpri*), the oscillator (*Vosc*). (**b**) Simulation curve of charging process. (**c**) Experiment curve of charging process. (**d**) Comparison to charge a capacitor via a rectifier bridge and conditioning circuit. (**e**) Maximum voltage of the storage capacitor and the required time. (**f**) When resistive load is connected, load voltage and output voltage of the primary inductance.

For a non-ideal coupling inductance, it has power loss, which increases as the frequency decreases. However, the frequency of TENG output voltage that depends on the environment vibration is always low. So, in the circuit, the bi-directional switch is used to increase the frequency so that the power loss of the coupling inductances could be reduced as much as possible. The control signal of bi-directional switch is needed. An oscillation circuit that can generate a signal at a certain frequency to control the bi-directional switch is essential. When considering the primary circuit, when the circuit is turned on, the current on the inductor cannot change immediately, there will be oscillation due to the step response, it will cause the induced voltage on the secondary coil. Then, damping oscillation voltage will be observed on the storing capacitor due to the resistance of the non-ideal coupling inductance. The storing capacitor charged step by step as show in Figure 3a,b. When the circuit is turned on, the electric energy is passed to the secondary coil through the coupling inductance, and according to the secondary load, there will be different attenuation coefficient, so the turn on time is essential to make sure energy can be passed out. However, the turn-off time determines initial voltage of the primary coil. So frequency of the control circuit is also an important parameter as shown in Figure 3e,f. In Figure 3e, a 10 μf capacitor is chosen as the storing capacitor, maximum voltage of the storage capacitor $V_{S}\left(max\right)$ gets a maximum value as the frequency of the control circuit range from 0.1 to 100 KHz. When the load is a 10 KΩ resistor, the peak load voltage $V_{r}$ and peak voltage of the primary inductance $V_{i}\left(max\right)$ are shown in Figure 3f.

The experimental results show that the self-powered management circuit can meet the voltage, and power consumption needs of a wireless sensor node. In experimental test, the maximum open circuit voltage of TENG is 83.6 V, frequency is 5 Hz, when the TENG starts working, the storing capacitor is charged. After about 3.6 s, the storing capacitor voltage is basically stable at 5.5 V, as shown in Figure 3c. When compared with charging though a rectifier bridge directly, the proposed circuit greatly reduces the charging time and increases efficiency by 50%, as shown in Figure 3d.

**Figure 4:**
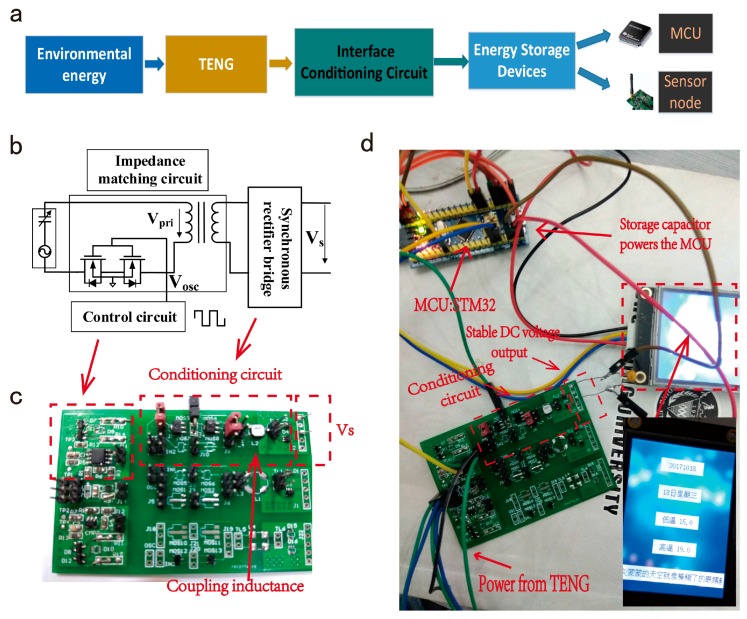
(**a**) An environmental energy harvesting system based on TENG. (**b**) The interface conditioning circuit for TENG. (**c**) Physical picture of the interface conditioning circuit. (**d**) An application of the environmental energy harvesting system based on TENG and the interface conditioning circuit.

Figure 4a shows a wireless sensor system based on environmental energy harvester by using TENG. The TENG transform environmental energy into electrical energy, then a stable DC voltage could be used though an interface conditioning circuit, so that it could power the wireless sensor node. The proposed circuit for the TENG is composed of an impedance matching circuit, a synchronous rectifier bridge, a control circuit, an energy storage device, as shown in Figure 4b. The whole PCB circuit is shown in Figure 4c. Using TENG to power a wireless sensor node which consists of a microcontroller unit (STM32, STMicroelectronics, Geneva, Swiss Confederation) via the interface conditioning circuit. The MCU (Microcontroller Unit) accesses the data, including weather information and the date, from the server through Wi-Fi and display on the screen successfully, as shown in Figure 4d.

## 4. Conclusions

This paper proposed an adaptable interface conditioning circuit, which is composed of an impedance matching circuit, a synchronous rectifier bridge, a control circuit, and an energy storage device. Especially, a novel bi-directional switch control mechanism was adopted to increase the frequency in order to reduce the energy loss of coupling inductance and increase the output efficiency of TENG. Experimental results show that, in about 3.6 s, the storing capacitor voltage was basically stable at 5.5 V by using the proposed adapted interface conditioning circuit in this paper. The charging efficiency has increased by 50%. The TENG’s application in a self-powered sensor will be promoted in the future by adopted this proposed adaptable interface conditioning circuit technology.
